# A Systemic Study of Subcellular Localization of Porcine Epidemic Diarrhea Virus Proteins

**DOI:** 10.3390/pathogens11121555

**Published:** 2022-12-17

**Authors:** Huixin Zhu, Zitong Li, Juan Bai, Ping Jiang, Xianwei Wang, Xing Liu

**Affiliations:** 1 Key Laboratory of Animal Disease Diagnostics and Immunology, Ministry of Agriculture, MOE International Joint Collaborative Research Laboratory for Animal Health & Food Safety, College of Veterinary Medicine, Nanjing Agricultural University, Nanjing 210095, China; 2 Jiangsu Co-Innovation Center for Prevention and Control of Important Animal Infectious Diseases and Zoonoses, Yangzhou 225000, China

**Keywords:** PEDV proteins, subcellular localization, endoplasmic reticulum (ER), Golgi apparatus, mitochondria

## Abstract

Porcine epidemic diarrhea virus (PEDV), a highly pathogenic enteric coronavirus, is regarded as one of the most severe porcine pathogens. To date, there are still no commercial vaccines or drugs that can provide full protection against the epidemic strains. A better understanding of the subcellular location of individual proteins could benefit from studying the protein functions and mechanisms of how the virus regulates key cellular processes, finally leading to the development of antiviral agents. In this study, we characterized the subcellular localization of PEDV proteins using multi-labeled fluorescent immunocytochemistry. As a result, 11 proteins showed cytoplasmic distribution and 10 proteins showed both cytoplasmic and nuclear distribution. Furthermore, we demonstrated that four proteins (Nsp3, Nsp4, Nsp6, and S1) were co-localized in the endoplasmic reticulum (ER), while four proteins (Nsp2, S2, N, and ORF3) were partially observed in the ER, two proteins (E and M) were co-localized in the Golgi apparatus, and two proteins (Nsp2 and E) were partially co-localized with the mitochondria. These viral proteins may perform specific functions at specific cellular locations. Together, these results describe a subcellular localization map of PEDV proteins, which will help to characterize the functions of these proteins in the future.

## 1. Introduction

**Figure 1:**
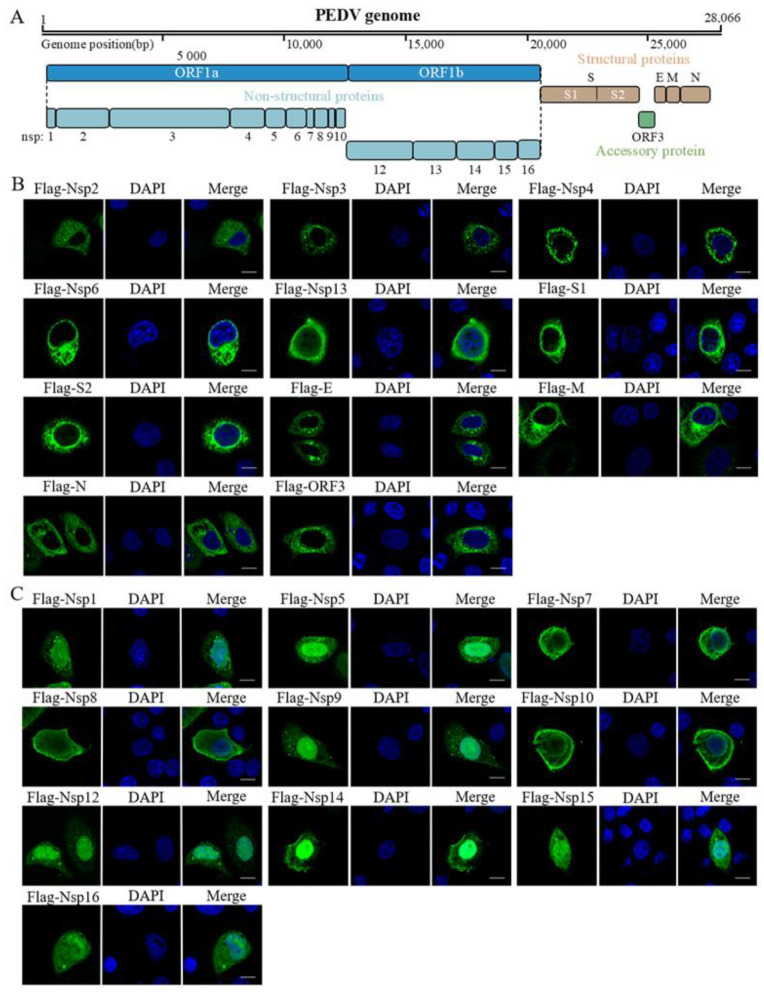
Genome annotation of PEDV and immunolocalization of Flag-tagged PEDV proteins. (**A**), the genome of MSCH has 28,066 nucleotides and encodes seven open reading frames responsible for approximately 21 proteins. (**B**,**C**) HeLa cells were transfected with Flag-tagged viral proteins. The IFA was carried out to assess the viral protein (CoraLite^®^488-conjugated secondary antibodies) using the anti-Flag antibody, and the cells were stained with DAPI to show the nucleus in blue. Scale bar = 10 μm.

Porcine epidemic diarrhea virus (PEDV) is a highly pathogenic enteric coronavirus that causes gastrointestinal disease and high mortality in piglets [1]. PEDV was first identified as the etiological agent of porcine epidemic diarrhea (PED) in England and Belgium in the 1970s [2,3], and it re-emerged in Asia in 2010 and resulted in enormous economic losses [4,5,6]. Similar to other coronaviruses, PEDV is an enveloped positive-sense single-stranded RNA virus [7]. The complete genome of PEDV is approximately 28 kb, encoding seven open reading frames (ORFs). As shown in Figure 1A, the two large ORFs, 1a and 1b, occupy almost two-thirds of the genome, encoding two large replicase proteins, which are post-translationally cleaved into 16 nonstructural proteins (Nsps), Nsp1–Nsp16. The remaining ORFs encode spike (S), envelope (E), membrane (M), and nucleocapsid (N) proteins and one accessory protein, ORF3 [8]. Most of these proteins play roles during viral replication. Nsp1 is encoded by the gene closest to the 5′ end of the viral genome and is among the first proteins to be expressed after cell infection to repress the innate immune response by inhibiting host protein expression [9].

The innate immune response forms the first line of host defense against invading pathogens [10]. However, many viruses, including coronaviruses, have evolved with diverse strategies to prevent the activation of antiviral effectors, particularly by inhibiting interferon (IFN) production. Of the 21 PEDV proteins, Nsp1, Nsp3, Nsp5, Nsp8, Nsp14, Nsp15, Nsp16, ORF3, E, M, and N suppress type III IFN activities [11]. Nsp3 is a papain-like proteinase protein that mediates the proteolytic processes of viral polyproteins, and it has been reported that PEDV replicase encoded papain-like protease 2 acts as a viral deubiquitinase negatively regulating the type I interferon pathway [12,13]. Nsp5, also named 3C-like protease, is able to hydrolyze some host cell proteins to evade immune response. PEDV nsp5 has been shown to cleave the NF-κB essential modulator to perform interferon antagonism [14]. It has also been reported that the Nsp7 of PEDV can inhibit the production of IFN-stimulated genes by inhibiting the JAK-STAT signaling [15]. The N protein suppresses IRF3 and NF-κB, and antagonizes IFN-*β* production [16].

Moreover, the S protein has a single transmembrane domain and serves as the attachment and fusion protein. The coronavirus E protein is a small membrane protein with ion channel function, which plays an important role in virion assembly, virus excretion and host stress response [17]. The M protein is the most abundant component of the viral envelope and plays required, key roles in virus assembly [18]. In addition, coexpression of the M and E proteins alone is reported to be sufficient for the virus-like particle assembly of most coronaviruses [19].

The limited viral genome and structure compel the virus to enlist host systems for its replication and transmission. In this process, the virus has also evolved pathways to avoid traps or self-degrade, and transports itself to the appropriate intracellular destination, where it can replicate and assemble. Diverse viruses, including RNA and DNA viruses, that use the luminal and membrane contents of the endoplasmic reticulum (ER) to achieve proper entry, replication, or assembly have evolved [20]. For instance, during infection, coronaviruses assemble by budding into the lumen of the intermediate compartment at the ER–Golgi interface [21]. The ER apparatus is also the factory for viral protein production; therefore, it is the main organelle manipulated by viruses. Viruses can also manipulate the mitochondria to help them complete their life cycle. In addition, viral proteins can interfere with mitochondrial membrane-associated proteins to disrupt functional responses, such as energy, protein folding, and redox homeostasis [22]. A previous study has indicated that the Nsp2 of SARS-CoV interacts with the mitochondrial inner membrane components PHB1 and PHB2, resulting in the disruption of intracellular host signaling [23].

The protein localization can provide important information regarding PEDV infection and virus–host interactions. Understanding how PEDV hijacks the host machinery and which of its proteins are key for its interaction will be crucial in identifying effective targets for therapeutic interventions.

## 2. Materials and Methods

### 2.1. Cell Lines, Plasmids and Antibodies

HeLa cells were maintained in Dulbecco’s modified Eagle’s medium (DMEM) (Gibco, Grand Island, NY, USA) supplemented with 10% fetal calf serum (Gibco, Grand Island, NY, USA) and penicillin (250 U/mL)–streptomycin (250 μg/mL). The cells were incubated at 37 °C in a humidified incubator with 5% CO2. The plasmids pmScarlet_Giantin_C1 (Plasmid #85048) for visualizing the Golgi body and mCherry-TOMM20-N-10 (Plasmid #55146) for visualizing mitochondria were purchased from Addgene. Anti-Calnexin (H-107, sc-11398) for visualizing ER was purchased from Abcam (Cambridge, UK). Anti-Flag antibody was purchased from Abmart (Shanghai, China).

### 2.2. Molecular Cloning

The sequence of PEDV (GenBank accession no. MT683617) isolate, MSCH, was used in this study for the DNA synthesis of each gene. We cloned all the viral genes (Nsp1–Nsp16, except for Nsp11, which is a small oligopeptide, S, M, N, E, and ORF3) encoded by the PEDV genome and separately inserted them into the pXJ41 vector fused with a Flag tag and expressed them in HeLa cells. Among them, the DNA sequences of Nsp2, Nsp3, Nsp4, Nsp6, and ORF3 were codon-optimized to obtain a high expression level in human cells. Full-length S cloning has not been successful; so, we expressed S1 and S2 according to their cleavage phase [24]. The cloning information about all PEDV genes is provided in Supplementary Table S1.

### 2.3. Immunofluorescence Assay

Immunostaining was performed on cells grown on a 15-mm cell culture dish (Nest Biotechnology, Wuxi, China) after fixation with 4% paraformaldehyde for 15 min at room temperature and permeabilization in 0.1% Triton X-100 for 10 min; then, the cells were sequentially incubated with anti-Flag and CoraLite^®^488-conjugated secondary antibodies (Proteintech, China) for 1 h each. Finally, the cells were stained with DAPI (Biosharp, Guangzhou, China) to show the nucleus in blue.

### 2.4. Confocal Microscopy

The cells were visualized using a confocal microscope (Nikon A1, Fujisawa, Japan). All the photomicrographs were taken at a magnification of 600×. Two or three channels were recorded sequentially or simultaneously while watching for potential overlaps among the different color signals.

## 3. Results

To reveal the subcellular location of the PEDV proteins, we transfected the indicated recombinant plasmid into HeLa cells. At 24 h post-transfection, the cells were fixed and immunostained with anti-Flag antibodies. The fluorescence signaling was detected to reflect the viral protein level. As shown in Figure 1, the PEDV-encoded proteins could be broadly classified into two groups: 11 proteins (Nsp2, Nsp3, Nsp4, Nsp6, Nsp13, S1, S2, E, M, N, and ORF3) showed cytoplasmic localization (Figure 1B) and 10 proteins (Nsp1, Nsp5, Nsp7, Nsp8, Nsp9, Nsp10, Nsp12, Nsp14, Nsp15, and Nsp16) were present in both the cytoplasm and the nucleus (Figure 1C). Next, based on their specific localization details, we explored the subcellular organelle localization of the proteins distributed in the cytoplasm.

**Figure 2:**
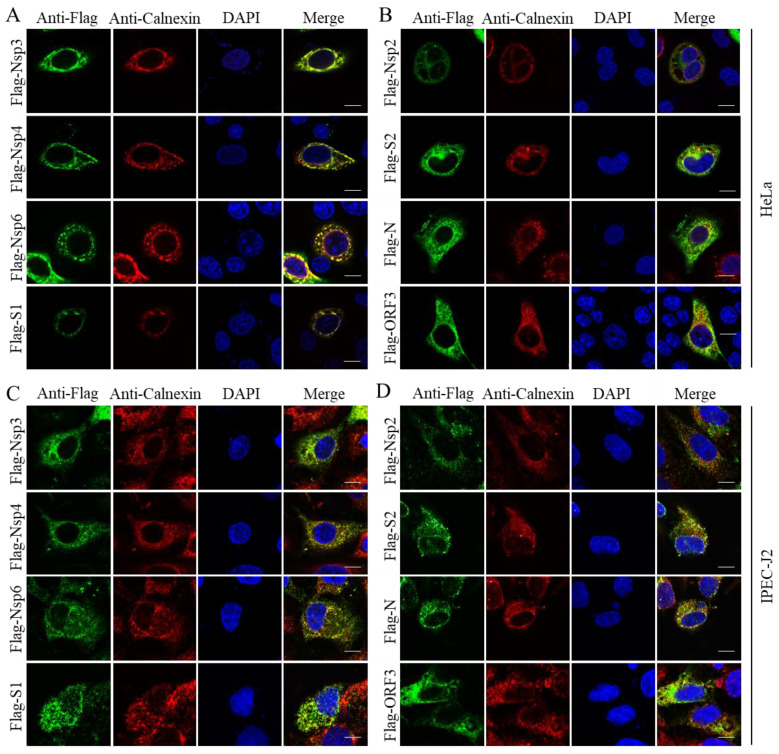
Nsp3, Nsp4, Nsp6, S1, S2, N, and ORF3 of PEDV are related to ER. The viral protein-expressing plasmids were transfected into HeLa and IPEC-J2 cells for 24 h, fixed, and immunostained with anti-Flag antibodies. The samples were also immunostained for the ER-localized protein, using anti-Calnexin and CoraLite^®^594-conjugated secondary antibodies (Proteintech, China). (**A**,**C**) Nsp3, Nsp4, Nsp6, and S1 co-localize in the ER. (**B**,**D**) Nsp2, S2, N, and ORF3 are partially located in the ER. Scale bar = 10 μm.

Increasing evidence suggests that the ER plays a critical role in supporting viral infection. To determine which proteins of the PEDV are located in the ER, we transfected the cytoplasmic viral proteins into HeLa and IPEC-J2 cells for 24 h, which were then stained as PEDV proteins with anti-Flag in green and ER apparatus with anti-Calnexin in red. Through the degree of overlap between the red and green fluorescence, we found that the viral proteins Nsp3, Nsp4, Nsp6, and S1 were co-localized in the ER (Figure 2A), which is consistent with those in SARS-CoV and MERS-CoV [25]. As shown in Figure 2B, Nsp2, S2, N, and ORF3 were partially co-localized in the ER. There is also some scattered green fluorescence in the Merge image in addition to the overlapping area, probably because these proteins need to perform different biological functions in different positions.

**Figure 3:**
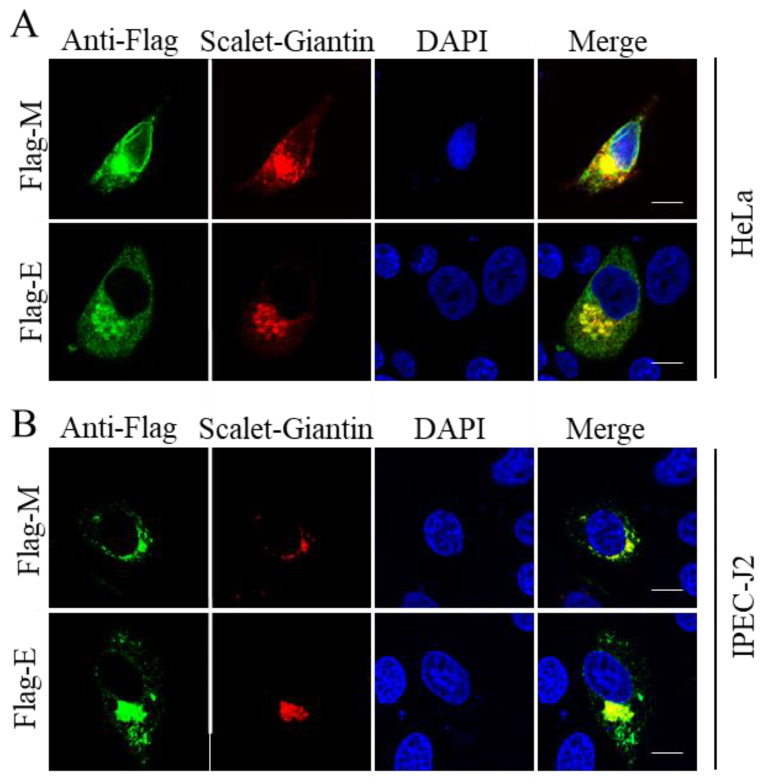
M and E are localized at the Golgi apparatus. HeLa (**A**) and IPEC-J2 (**B**) cells were co-transfected with Flag-tagged viral proteins and Golgi apparatus protein-expressing plasmid pmScarlet_Giantin_C1 (Scarlet-Giantin), and then fixed and stained; the viral proteins show green fluorescence, and the Golgi apparatus shows red fluorescence. Scale bar = 10 μm.

The Golgi apparatus is an organelle that plays important roles in cellular homeostatic functions. Importantly, recent studies have shown that the Golgi apparatus is associated with different possible pathways for the egress of coronaviruses [26]. Next, we examined the co-localization of viral proteins with the Golgi apparatus. The plasmid-encoded viral proteins and Golgi apparatus proteins (pmScarlet_Giantin_C1) were co-transfected into HeLa and IPEC-J2 cells, which were then fixed 24 h post-transfection and stained; the viral proteins were stained with green fluorescence by the anti-Flag antibodies, and the Golgi apparatus show red fluorescence because the expression plasmid is tagged with scarlet. As shown in Figure 3, the E and M proteins co-localized with the Golgi apparatus. This result is consistent with that obtained for other coronaviruses [27,28], the E and M proteins of PEDV are also involved in virus assembly and maturation.

**Figure 4:**
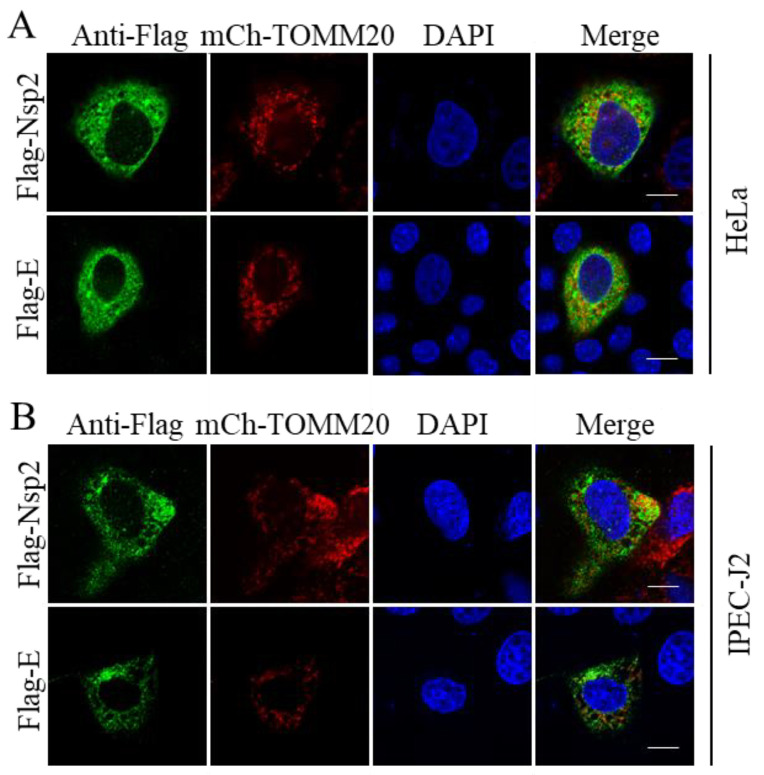
Nsp2 and E are associated with the mitochondria. HeLa (**A**) and IPEC-J2 (**B**) cells were co-transfected with Flag-tagged viral proteins and mitochondrial protein-expressing plasmid mCherry-TOMM20-N-10 (mCh-TOMM20), fixed 24 h post-transfection, and then immunostained; the viral proteins show green fluorescence and the mitochondria show red fluorescence. Scale bar = 10 μm.

Mitochondria play an important role in the response of the host to viral infection and immunity [29]. Therefore, we probed whether there are viral proteins that interact with the mitochondria. For this purpose, several viral-protein-expressing plasmids were separately transfected together with mCherry-TOMM20-N-10, and the latter is a well-known mitochondrial protein [30]. The IFA results show that Nsp2 and E are partially co-localized with the mitochondria (Figure 4). Thus, our results suggest that these proteins may play their biological functions through interaction with the mitochondria.

## 4. Discussion

To understand the detailed function of PEDV-encoded proteins, we expressed all the viral proteins in HeLa cells to determine their subcellular localization map. We found a diversity of protein distribution in the cells, suggesting a complicated interaction of PEDV with host cells to achieve a successful infection. A summary of all the results is presented in Table 1, and there is a correlation between our results and those of previous studies. We found that all structural proteins and accessory protein ORF3 are positioned in the cytoplasm. Of the 16 non-structural proteins, Nsp1, Nsp5, Nsp7, Nsp8, Nsp9, Nsp10, Nsp12, Nsp14, Nsp15, and Nsp16 have nuclear localization and may function in the nucleus. For example, PEDV Nsp1 inhibits NF-κB activity and causes degradation of the CREB-binding protein in the nucleus, which may be related to its nuclear-like distribution [31,32].

Subcellular localization of a protein reflects its function and provides insights into its potential contribution during viral infection. The ER is the primary organelle for viral protein production; viruses must use it for viral replication. In SARS-CoV, Nsp3, Nsp4, and Nsp6 have been reported to induce double-membrane vesicles during SARS-CoV infection, which is essential for viral replication [33]. The results of this study showed that Nsp3, Nsp4, and Nsp6 of PEDV colocalized with the ER, so we speculate that these three proteins of PEDV may also have the same function as demonstrated in SARS-CoV. This phenomenon reminds us that we can use some methods to disrupt the co-localization of these three proteins and thus inhibit the replication of PEDV. In addition, it has been reported that deletion of the ER retrieval signal of the S protein of PEDV and SARS-CoV can reduce the cell surface expression of S, hence affecting the rate of syncytia formation [34,35]. Previous studies have suggested that both PEDV N and ORF3 can cause ER stress to exercise their functions [36,37]. Collectively, the relationship between ER and these proteins might be important for PEDV replication, which remains to be investigated. Moreover, the Nsp2 of SARS-CoV-2 co-localizes with the ERLIN1/2 complex that regulates mitochondrial function and calcium flux [33], and further studies are required to determine whether the Nsp2 of PEDV has a similar function.

In summary, we cloned all the proteins of PEDV into expressing vectors and assessed their subcellular localization using selected markers for subcellular organelles. Different location of PEDV proteins in host cells suggests a dynamic incorporation of each viral protein during PEDV assembly, trafficking, maturation, and production. Knowledge of the cellular localization pattern of individual PEDV proteins could help infer their biological function based on the processes available in the cellular compartment. Our findings provide new insights and references into the biological function of PEDV proteins. Further research is required using protein-specific antibodies to reflect the real situation of viral protein localizations under infections.

## Figures and Tables

**Table 1:** Characterization of PEDV proteins.

Name	Cellular/Subcellular Location
Nsp1	Cyto. & Nuc.
Nsp2	Cyto., Endoplasmic reticulum, Mitochondria
Nsp3	Cyto., Endoplasmic reticulum
Nsp4	Cyto., Endoplasmic reticulum
Nsp5	Cyto. & Nuc.
Nsp6	Cyto., Endoplasmic reticulum
Nsp7	Cyto. & Nuc.
Nsp8	Cyto. & Nuc.
Nsp9	Cyto. & Nuc.
Nsp10	Cyto. & Nuc.
Nsp12	Cyto. & Nuc.
Nsp13	Cyto.
Nsp14	Cyto. & Nuc.
Nsp15	Cyto. & Nuc.
Nsp16	Cyto. & Nuc.
S1	Cyto., Endoplasmic reticulum
S2	Cyto., Endoplasmic reticulum (partially)
E	Cyto., Golgi apparatus, Mitochondria
M	Cyto., Golgi apparatus
N	Cyto., Endoplasmic reticulum (partially)
ORF3	Cyto., Endoplasmic reticulum (partially)

Cyto: Cytoplasm; Nuc: Nucleus.

## Data Availability

Not applicable.

## Supplementary Materials

The following supporting information can be downloaded at: https://www.mdpi.com/article/10.3390/pathogens11121555/s1. Table S1: The cloning information on all PEDV genes.

## References

[1] Jung K., Saif L.J. (2015). Porcine epidemic diarrhea virus infection: Etiology, epidemiology, pathogenesis and immunoprophylaxis. Vet. J..

[2] Wood E.N. (1977). An apparently new syndrome of porcine epidemic diarrhoea. Vet. Rec..

[3] Pensaert M.B., de Bouck P. (1978). A new coronavirus-like particle associated with diarrhea in swine. Arch. Virol..

[4] Li W., Li H., Liu Y., Pan Y., Deng F., Song Y., Tang X., He Q. (2012). New Variants of Porcine Epidemic Diarrhea Virus, China, 2011. Emerg. Infect. Dis..

[5] Sun R., Cai R., Chen Y., Liang P., Chen D., Song C. (2012). Outbreak of porcine epidemic diarrhea in suckling piglets, China. Emerg. Infect. Dis..

[6] Lee S., Lee C. (2014). Outbreak-related porcine epidemic diarrhea virus strains similar to US strains, South Korea, 2013. Emerg. Infect. Dis..

[7] Song D., Park B. (2012). Porcine epidemic diarrhoea virus: A comprehensive review of molecular epidemiology, diagnosis, and vaccines. Virus Genes.

[8] Duarte M., Gelfi J., Lambert P., Rasschaert D., Laude H. Genome Organization of Porcine Epidemic Diarrhea Virus. Proceedings of the 5th International Symposium on Coronaviruses.

[9] Kamitani W., Narayanan K., Huang C., Lokugamage K., Ikegami T., Ito N., Kubo H., Makino S. (2006). Severe acute respiratory syndrome coronavirus nsp1 protein suppresses host gene expression by promoting host mRNA degradation. Proc. Natl. Acad. Sci. USA.

[10] Takeuchi O., Akira S. (2010). Pattern recognition receptors and inflammation. Cell.

[11] Zhang Q., Ke H., Blikslager A., Fujita T., Yoo D. (2018). Type III Interferon Restriction by Porcine Epidemic Diarrhea Virus and the Role of Viral Protein nsp1 in IRF1 Signaling. J. Virol..

[12] Yang X., Chen X., Bian G., Tu J., Xing Y., Wang Y., Chen Z. (2014). Proteolytic processing, deubiquitinase and interferon antagonist activities of Middle East respiratory syndrome coronavirus papain-like protease. J. Gen. Virol..

[13] Xing Y., Chen J., Tu J., Zhang B., Chen X., Shi H., Baker S., Feng L., Chen Z. (2013). The papain-like protease of porcine epidemic diarrhea virus negatively regulates type I interferon pathway by acting as a viral deubiquitinase. J. Gen. Virol..

[14] Wang D., Fang L., Shi Y., Zhang H., Gao L., Peng G., Chen H., Li K., Xiao S. (2016). Porcine Epidemic Diarrhea Virus 3C-Like Protease Regulates Its Interferon Antagonism by Cleaving NEMO. J. Virol..

[15] Zhang J., Yuan S., Peng Q., Ding Z., Hao W., Peng G., Xiao S., Fang L. (2022). Porcine Epidemic Diarrhea Virus nsp7 Inhibits Interferon-Induced JAK-STAT Signaling through Sequestering the Interaction between KPNA1 and STAT1. J. Virol..

[16] Ding Z., Fang L., Jing H., Zeng S., Wang D., Liu L., Zhang H., Luo R., Chen H., Xiao S. (2014). Porcine epidemic diarrhea virus nucleocapsid protein antagonizes beta interferon production by sequestering the interaction between IRF3 and TBK1. J. Virol..

[17] Cao Y., Yang R., Lee I., Zhang W., Sun J., Wang W., Meng X. (2021). Characterization of the SARS-CoV-2 E Protein: Sequence, Structure, Viroporin, and Inhibitors. Protein Sci. Publ. Protein Soc..

[18] De Haan C., Vennema H., Rottier P. (2000). Assembly of the coronavirus envelope: Homotypic interactions between the M proteins. J. Virol..

[19] Vennema H., Godeke G., Rossen J., Voorhout W., Horzinek M., Opstelten D., Rottier P. (1996). Nucleocapsid-independent assembly of coronavirus-like particles by co-expression of viral envelope protein genes. EMBO J..

[20] Den Boon J.A., Ahlquist P. (2010). Organelle-Like Membrane Compartmentalization of Positive-Strand RNA Virus Replication Factories. Annu. Rev. Microbiol..

[21] Krijnselocker J., Ericsson M., Rottier P.J.M., Griffiths G. (1994). Characterization of the budding compartment of mouse hepatitis virus—Evidence that transport from the RER to the Golgi complex requires only one vesicular transport step. J. Cell Biol..

[22] Chatel-Chaix L., Cortese M., Romero-Brey I., Bender S., Neufeldt C.J., Fischl W., Scaturro P., Schieber N., Schwab Y., Fischer B. (2016). Dengue Virus Perturbs Mitochondrial Morphodynamics to Dampen Innate Immune Responses. Cell Host Microbe.

[23] Cornillez-Ty C.T., Liao L., Yates III J.R., Kuhn P., Buchmeier M.J. (2009). Severe Acute Respiratory Syndrome Coronavirus Nonstructural Protein 2 Interacts with a Host Protein Complex Involved in Mitochondrial Biogenesis and Intracellular Signaling. J. Virol..

[24] Peng G., Sun D., Rajashankar K.R., Qian Z., Holmes K.V., Li F. (2011). Crystal structure of mouse coronavirus receptor-binding domain complexed with its murine receptor. Proc. Natl. Acad. Sci. USA.

[25] Gordon D.E., Hiatt J., Bouhaddou M., Rezelj V.V., Ulferts S., Braberg H., Jureka A.S., Obernier K., Guo J.Z., Batra J. (2020). Comparative host-coronavirus protein interaction networks reveal pan-viral disease mechanisms. Science.

[26] Prydz K., Saraste J. (2022). The life cycle and enigmatic egress of coronaviruses. Mol. Microbiol..

[27] Arndt A.L., Larson B.J., Hogue B.G. (2010). A Conserved Domain in the Coronavirus Membrane Protein Tail Is Important for Virus Assembly. J. Virol..

[28] Klumperman J., Locker J.K., Meijer A., Horzinek M.C., Geuze H.J., Rottier P.J.M. (1994). Coronavirus M proteins accumulate in the Golgi complex beyond the site of virion budding. J. Virol..

[29] Koshiba T. (2013). Mitochondrial-mediated antiviral immunity. Biochim. Biophys. Acta Mol. Cell Res..

[30] Goping I.S., Millar D.G., Shore G.C. (1995). Identification of the human mitochondrial protein import receptor, huMas20p—Complementation of Δmas20 in yeast. Febs Lett..

[31] Zhang Q., Ma J., Yoo D. (2017). Inhibition of NF-κB activity by the porcine epidemic diarrhea virus nonstructural protein 1 for innate immune evasion. Virology.

[32] Zhang Q., Yoo D. (2016). Immune evasion of porcine enteric coronaviruses and viral modulation of antiviral innate signaling. Virus Res..

[33] Davies J., Almasy K., McDonald E., Plate L. (2020). Comparative Multiplexed Interactomics of SARS-CoV-2 and Homologous Coronavirus Nonstructural Proteins Identifies Unique and Shared Host-Cell Dependencies. ACS Infect. Dis..

[34] Hou Y., Meulia T., Gao X., Saif L.J., Wang Q. (2019). Deletion of both the Tyrosine-Based Endocytosis Signal and the Endoplasmic Reticulum Retrieval Signal in the Cytoplasmic Tail of Spike Protein Attenuates Porcine Epidemic Diarrhea Virus in Pigs. J. Virol..

[35] Cattin-Ortola J., Welch L.G., Maslen S.L., Papa G., James L.C., Munro S. (2021). Sequences in the cytoplasmic tail of SARS-CoV-2 Spike facilitate expression at the cell surface and syncytia formation. Nat. Commun..

[36] Zou D., Xu J., Duan X., Xu X., Li P., Cheng L., Zheng L., Li X., Zhang Y., Wang X. (2019). Porcine epidemic diarrhea virus ORF3 protein causes endoplasmic reticulum stress to facilitate autophagy. Vet. Microbiol..

[37] Xu X., Zhang H., Zhang Q., Huang Y., Dong J., Liang Y., Liu H.-J., Tong D. (2013). Porcine epidemic diarrhea virus N protein prolongs S-phase cell cycle, induces endoplasmic reticulum stress, and up-regulates interleukin-8 expression. Vet. Microbiol..

